# The Effects of HIV/AIDS Scourge on Production and Income among Rural Households in Adamawa State of Nigeria

**DOI:** 10.5539/gjhs.v4n1p245

**Published:** 2012-01-01

**Authors:** Iya I. B., Purokayo G.S., Gabdo Yusuf

**Affiliations:** Department of Economics Federal University of Technology, Yola, Nigeria

**Keywords:** Labour force participation, HIV/AIDS, AIDS scourge, Household productivity

## Abstract

The paper investigates the determinants and the impact of HIV/AIDS on households in Adamawa State. 120 respondents affected with HIV/AIDS were selected for interview using simple random sampling techniques. Both primary and secondary data were used in its analysis to determine the impact of the disease on household’s income. The data collected were analyzed using descriptive analytical techniques and a logistic regression model was employed to estimate the likelihood that a household witnessed a fall in income as a result of the disease. The paper revealed that HIV/AIDS had an adverse impact on household’s productivity, income, saving and capital formation. The paper, therefore recommends an intensive Aids education programme and Government at all level as well as NGO’s should endeavor to provide adequate HIV testing kits, medication and free counseling services to enable the households determine their HIV status.

## 1. Introduction

The health status of the people in any country is a critical element in its economic growth as well as an important factor in the overall quality of household’s life and productivity. AIDS tend to selectively affect women, youths, which are the vulnerable groups; it influences a wide range of social and economic factors. In Adamawa State, agriculture is the dominant industry in the state, thus any serious infection of HIV could lead to low productivity of the industry. Recent studies have revealed that HIV/AIDS prevalence among women and youths is increasingly worrisome. The target is the most productive group of the population (15-49 groups). This is the age bracket involved in social and economic sectors as well as the civil service and education. Thus these sectors could suffer severely from labor supply, loss of time due to illness, funeral bills, orphans, street children, medical expenditure and other vices. The consequences could be disastrous if nothing is done to control the spreads.

National prevalence has been steadily increasing from 1.8 percent in 1990 to 5.5 percent in 1999. The difference between HIV seroprevalence in urban and rural areas is not large, indicating that the AIDS problem in Nigeria is not strictly an urban one. This trend dictates labour participation rates both in the rural Nigeria; where the labour force is engaged in agricultural production, and in Urban Nigeria where most Nigerians migrate, find information to travel abroad for so called better life.

Factors that determine the labour force participation are the size of the family, level of education, job opportunities and migration amongst others. These are the main demographic dynamics in Nigeria over the years. Demographic indicators show that the dynamics in the population are skewed towards low productivity and increased social problems – high population movements to cities (Urban), high HIV/AIDS infections, and porous borders of the study area (Adamawa State) with Cameroun and Niger resulting to increased congestions and the rate infections.

Labour is total human efforts used in the productive process. It can also be defined as the strength expanded on the creation of goods and services. It includes number of hours worked and the physical strength. Labour can be services that have value and exchange. Economics is therefore concerned with efficiency – best use of productive resources and value. This is why policies for enhancing the labour force productivity is vital in achieving growth goals of the Nigerian economy, for instance the Vision 2020, which depended on the quality of the labour force, that will propel this economy to the desired growth by the year 2020.

The main objective of this paper is to examine the effects of HIV/AIDS scourge on labour force productivity of Adamawa state households.

## 2. Problem Statement

The health status of the people in any country is a critical element in the growth of its economy as well as an important factor in the overall quality of life. AIDS tend to selectively affect women, youths, which are the vulnerable groups; it will influence a wide range of social and economic factors. In Adamawa State, agriculture is the dominant industry; high rate of infection could lead to the stagnation of the industry. Recent studies have revealed that HIV/AIDS prevalence among women and youths is increasingly worrisome. The target is the most productive group of the population (15-49 groups).

## 3. Literature Review and Conceptual Framework

Until the early 1990’s, the empirical economic growth literature focused exclusively on the role of capital and labour (The later often augmented by schooling and technological change, but hardly ever on health as a key element of human capital (Baro, 1991). Even where a relationship has been found between indicators of health and income per capita, it has either been discounted or thought to be an indication of the impact of economic development on health. The standard perspective of this earlier literature appears to have been that of Preston (1976) who noted that key role of economic development in improving life expectancy.

Studies carried out by UNAID and ECA in 2003 revealed that HIV/AIDS appear to be having devastating effects on the economies of East and South African countries. Evidence from these studies shows that: (a). Millions of children are orphaned by AIDS; and the number is growing. (b). Poverty is intensifying and deepening. (c). Productive capacity is being reduced in all sectors such that:-

- Food crises of 2003 in South Africa could be traced to HIV/AIDS infection.
- Depriving those economies scarce skills, and children of their parents. In addition, it is observed that HIV/AIDS could leave the Kenyan economy one-sixth smaller than it would be without a high prevalence by the year 2015. And in Southern Africa, by the year 2020, the level of GDP could be lowered by 17% due to HIV/AIDS while the level of per capita GDP could be lowered by 17%.

An empirical study of this nature is therefore necessary in an attempt to determine the actual effect of HIV/AIDS on the household’s productivity, income and standard of living. The objectives of the paper are therefore to assess the impact of HIV/AIDS on household in Adamawa State.

However, there is now significant evidence demonstrating the aggregate impact of health on growth and on level levels of real GDP per capita (Bhargava, 2001).

Bloom, Canning and Sevilla (2004) found that a one year improvement in the population’s life expectancy (a standard measure of health status) contributions to a 4% increase in output. In another study, the same authors estimate that a one percentage point increase in adult survival rates boosts labour productivity by about 2.8% formal analysis suggest that a country can, on average, expect to see per capita income grow by an extra 0.3 – 0.5% points a year for every 5 years it adds to its life expectancy. This is a considerable boost, given that between 1965 and 1990 global income per capita grew by an average of 2.0% per year (Bloom, Canning and Malaney, 2000).

Similar studies have been done in Asia by Andrew and Mason (2002) – that the effects of controls for initial income, (developing Asian) countries with infant mortality rates (Mason, 2002). The potential returns to health investment he concludes appear to be substantial in the region.

Moreover, studies that consider full income which assigns economic value to changes in life expectancy-suggest that falling mortality rates have a more substantial positive impact on economic development than is shown by GDP per capita data. For example, in a assessment of the growth of real income per capita on the United States over the 20^th^ century, (Nordhaus, 2003) concluded that over half of the growth in full income up to 1950 was attributable to mortality decline.

Considering the effect of mortality rates on full income suggest that estimates of the impact of AIDS on economic performance are under stated.

Bloom, Canning and Jamison (2004) suggest a new review of the literature on “value of statistical life (VSL) indicators that the adverse economic impact of AIDS in sub-Saharan Africa has already been more significant than GDP per capita data indicate.

Health affects labour productivity. Healthier workers are more energetic, have better attendance records and are likely to have higher mental capacity and morale. In development countries in particular, manual work makes up a large production of output, and physical endurance and strength rely crucially on sound health. A major study by Weil (2001) estimated that health differentials accounted for 17% of the difference in workers productivity between countries giving health roughly the same influence on productivity as physical capital (8%) and education (21%). Several micro-economic studies support this finding Strauss and Thomas (1998). The second channel from health to wealth involves the effect of health on education on education. Healthy children are better able to attend school and learn, and have more to gain by doing so because they can expect to live and work longer; and healthy families impose fewer burdens on children of having to care for sick relatives. An extra year of life expectancy is estimated to increase schooling levels by 0.25 years (Bill and Klenow, 2000).

Health improvements therefore spur the increase in savings which, by enabling greater investments in physical capital, spurs economic growth. East Asia’s dramatic savings boom between 1950 to 1990, which contributed greatly to its unprecedented economic growth (Bloom *et al*, 2004) was driven by the region’s rapidly improving life expectancy and by the increased production of people in the age groups that save the most (Lee, *et al*. 2000).

The household impact begins as soon as the member of a household starts suffering from AIDS related diseases. In addition to social and psychological consequences, three kinds of economic impacts can be distinguished. The first is the loss of income of the family members, in particular if he or she is the breadwinner. The second impact is the increase in household expenditures to cover the medical costs. The third impact is the indirect cost resulting from the absenteeism of members of the family from work or school to care for Aids patients.

The household impact begins as soon as a member of a household starts suffering from AIV/AIDS related disease. In addition to social and psychological consequences, three kinds of economic impacts can be distinguished. The first is the loss of income of the breadwinner. The second impact is the increase in household expenditures to cover the medical costs. The third impact is the indirect costs resulting from absenteeism of the members of the family from work or school to care for the aids patients.

A study in the same location (Adamawa State) by *Abdulazeez Abubarkar, Alo*

*Eammanuel and Naphthali Rebecca (2008)* found that, “*In spite of the persistent war against HIV/AIDS world all over, an amazing prevalence rate (14.2%) of the infection was still recorded in this part of the globe comprising of 1%, HIV-1, 0.51% HIV-2 and 1.6%, HIV-1+2 serotypes”*. This shows the degree of devastation of the disease in the state and its consequent effects on productivity of the state, showing its impacts on the working population over time. This has been manifested in low agricultural productivity of the state compared to the productivity of the sector in the 1980s and early 1990s.

## 4. Methodology

**Study Area:** The study was conducted in Adamawa State. The state is located in the North Eastern region of Nigeria. The study area was chosen in the North-Eastern Geo-political zone and export to other parts of the country, Central Africa, Niger and Federal Republic of Cameroon. The state has a total human population of 3, 194, 781 (NPC, 2006). The state has a total land area of 38, 741. 12 square kilometers (Uyang, 1993 and Tukur and Ardo, 1999). Out of these, the lowland, the hills and mountain ranges and upland plains constitute 32. 58%, 26.56% and 40.58 respectively (Tukur and Ardo, 1999). The state has (21) local government areas. Geographically, Adamawa State lies between latitude 7° and 11° and longitudes 11° and 14° (Adebayo, 1999). It share boundary with Taraba State in the South and West, Gombe in the North West and Borno State to the North. The state has an international boundary with Cameroon Republic along its eastern state.

The study was conducted between the periods of January 2010 to June 2010. The study covered six (6) local government areas out of 21 local government areas in the state. Two local governments were randomly selected from each of the three senatorial zones. And from each local government, four (4) villages were randomly selected, making a total of 24 villages sampled from the state. Five (5) respondents were also randomly picked from each village, making a total of one hundred and twenty (120) respondents to be randomly selected for the study. Data are obtained from both primary and secondary sources. While the former includes direct interviews with concerned individuals and families and the use of questionnaires, the latter encompasses hospitals and medical laboratories records from ten (10) major centres in the state. There are, however, series of HIV/AIDS related cases that were/are not recorded, for there is a very high tendency of several non-hospital HIV/AIDS issues, due to reasons peculiar to and inherent in rural communities.

The paper adopts a descriptive analytical technique resulting in evaluating the impact of HIV/AIDS on the households in the state.

## 5. Model Specification

A logistic regression model was employed to investigate the livelihood that household’s income level falls as a result of HIV/AIDS. The logistic regression model is as follows:


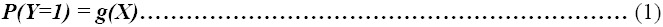


Where ***P (Y=1)*** is the probability that a household’ income level falls as a result of the disease.

***X*** is vector of the determinants of the odds of income level. The regression equation was build up by including variables that were presumed to affect the household poverty status. Such as:

(1) Sex of the household (Female=1 and Male=0),
(2). Household size.
(3). The sales of household assets which takes the value of 1 if the household sold the property and 0 if it did not.
(4). Total amount of money spend on caring for AIDS patients; on funeral and on other mourning expenses.

## 6. Result, Discussion and Analysis

Majority of the HIV/AIDS patients interviewed were males (61.7%), while only 38.3% of the respondents were females as shown on Table 1.0.

**Table 1:** Socio-economic characteristics of the respondents

CHARACTERISTICS	FREQUENCY	PERCENTAGE (%)
**Sex**		
Male	74	61.7
Female	46	38.3
*Total*	120	100
**Marital Status**		
Single	13	10.8
Married	70	58.3
Divorced/Separation	17	14.
Widow	20	16.7
*Total*	120	100
**Age Range**		
20-29	22	18.3
30-39	62	51.7
40-49	37	26.7
50 and above	4	1.3
*Total*	120	100
**Family Size**		
1-3	22	18.3
4-6	43	35.5
7-10	34	28.4
11 and a above	21	17.5
*Total*	120	100
**Years of Infection**		
1 – 5	110	91.7
6 – 10	6	5
11 – 15	4	3.3
16 and above	0	0.0
*Total*	120	100
**Educational Status**		
No formal education	70	58.33
Primary	30	25.00
Secondary	14	15.83
ND/University	1	0.08
*Total*	120	100

Source: Field Survey, 2010

It could be concluded that most of the HIV/AIDS patients in the study area are males. The study also shows that (58.3%) of the 120 respondents sampled were married, 10.5% were not married. And 14.2% and 16.7% of the respondents were divorcees and widows respectively.

Most of the HIV/AIDS patients are married and within the ages of 30 – 39 years (51%). More so, most of the sampled respondents have 4 – 6 persons per household, 91% were with the disease from 1– 5 years and most of them (58.3%) did not attend formal education.

Table 2.0 reveals that the pulled monthly labour and their values in maintaining and caring for AIDS patience. It shows that from the sampled of 120 respondents 120,500 man-hours were pulled from active labour force, while 555,700 man-hour were also pulled from those caring for them which would have been economically used productively.

**Table 2:** Analysis of value pulled monthly man-hours of labour to Aids

ITEM	HIV/AIDS patient man-hr.	CARING MEMBERS OF HOUSEHOLD: (man-hr)
Total man-hour lost to AIDS	120500	555.700
Average man-hour lost to AIDS	1004.167	4630.83
Average unit price of man-hour in Naira	150.00	150.00
Gross valued of pulled man-hour in Naira	18,075,000.00	83,355,00.00
Average valued of pulled man-hour in Naira	15,062.05	69,462.5

Source: Analysis of Field Survey Data, 2010

On the average, unit price per man-hour in N150.00 and the value pulled man-hours for the respondents per months is N15, 062.05 and for those caring for them, the estimated value stood at N69, 462.5

It can therefore be concluded that the man-hour and income lost to AIDS are tremendously. Hence, the income lost would have been saved or invested that in capable of improving the per capita income and standard of the hiring of the household in the state. The finding is in line with that of Mason (2004) who also confirmed that a lot of income has been lost away because of AIDS infection

This finding also agrees with (Dauda and Shuiabu, 2006) who positioned that HIV/AIDS may result in absence from work, loss of income temporary and if the patient dies the temporary loss income becomes a permanent loss.

The analysis in table 3.0 above reveals that many as 65% of the respondents in the households affected with HIV/AIDS attested experiencing a significant reduction in production and income as well. It was also observed that 82.0% said their tangible portion of their spending go on medication of the members affected with the disease in their households. Most of the respondents attested selling their assets ranging from jewelleries to landed property to coup with the cost of living and medication. About 57.0% of the respondents experienced reduction in quantity and quality of food consumption. Some of the respondents (58.0%) were compelled to adopt orphans within the extended family after the death of the parents. This may exert extra pressure on the affected families and which might be responsible for (56.0%) withdrawal of students from school.

**Table 3:** Effects of HIV/AIDS scourge o rural household

Effects	Yes (%)	No (%)
Reduction in production income	78(65.0)	42(35.0)
Spending of household saving/income on medical care	99(82.5)	21(17.5)
Selling of household assets to meat cost of living	84 (70.0)	36(30.0)
Reduction in quality and quantity of food consumption	68(56.67)	52(43.33
Adoption of orphans	58(48.33)	

Source: Field survey, 2011

The result table 4.0 reveals that HIV/AIDS contribute significantly to a reduction in household’s income. A large household is more prone to poverty than smaller one because HIV/AIDS diminishes a household’s ability to produce which more mouths to feed. Even when labor for participating in income generating activities is available, much of it is devoted to caring for the patients and less to production. More so, larger households are more likely to be poor; this may imply that the households of small size spend less on food and other materials than larger household.

**Table 4:** Logistic regressed results of income status among households with AIDS

Variables	Coefficients	Std error	Wald	P-value	Exp(B)	95% CI
**Constant**	-13.629	5.410	6.348	.012	0.00	
**Household Size (Hs)**	1.049	0.395	7.064	0.008	2.854	1.3176.184
**Sex of head of household (Shh)**	2.060	0.845	5.949	0.015	7.845	1.49841.073
**Sales of household assets (Sha)**	1.381	0.806	2.939	0.087	3.979	0.82019.31
**Total spending on Medicare (Tsm)**	0.785	0.408	3.699	0.054	2.191	0.9854.875

Wald Chi-square= 49.669 Pseudo R-squared=0.484(p-value= 0.000) Log likelihood=-47.139 Source: Analysis of field survey, 2010

Also the result shows that female headed households are more likely to have fall in income due to AIDS infection since men may be have more access to productive resources such as land credits technology, job opportunities etc. than female headed. Another explanation is that widows may themselves be infected and lacking the physical strength to engage effectively in income generating activities and food production. The result also revealed that household sale of family assets to cope with HIV/AIDS tends to reduce income levels of households.

Conclusively, from the analysis above, HIV/AIDS significantly reduces household income.

## 7. Conclusion

HIV/AIDS scourge has serious adverse effects on the household productivity, savings and capital accumulation and investments in Adamawa State as confirmed by study. Findings of the study reveal that HIV/AIDS has made significant contributions in reducing household income especially for female headed households. Further, the results revealed that the scourge has been responsible for loss of income, increased household expenditure for medical, funeral related expenses and a greater fall of household savings and assets.

## 8. Recommendations

An intensive HIV/AIDS education programme among the Nigeria populace is desirable and recommended. Government at all levels as well as NGO’s should provide adequate HIV/AIDS testing kits and free counseling services to enable every household to determine their status and consequently take necessary steps to prevent the spread and manage those already affected provision of affordable anti-retroviral drugs to the already affected households can go a long way in prolonging their lives as well as enhance their productivity.

The government design and implement effective, appropriate policies and programmes for poverty alleviation, targeting HIV/AIDs victims.
